# The Misattributed and Silent Causes of Poor COVID-19 Outcomes Among Pregnant Women

**DOI:** 10.3389/fmed.2021.745797

**Published:** 2021-10-26

**Authors:** Yossy Machluf, Sherman Rosenfeld, Izhar Ben Shlomo, Yoram Chaiter, Yaron Dekel

**Affiliations:** ^1^Unit of Agrigenomics, Shamir Research Institute, Haifa University, Kazerin, Israel; ^2^The Department of Science Teaching, Weizmann Institute of Science, Rehovot, Israel; ^3^Emergency Medicine Program, Zefat Academic College, Safed, Israel; ^4^The Israeli Center for Emerging Technologies in Hospitals and Hospital-Based Health Technology Assessment, Shamir (Assaf Harofeh) Medical Center, Zerifin, Israel; ^5^Department of Medical Laboratory Sciences, Zefat Academic College, Safed, Israel

**Keywords:** COVID-19, silent hypoxia, pregnancy, dyspnea, pulse oximetry, diagnosis and treatment

## Abstract

Abundant evidence strongly suggests that the condition of pregnancy makes women and their fetuses highly vulnerable to severe Corona-virus 2019 (COVID-19) complications. Here, two novel hypoxia-related conditions are proposed to play a pivotal role in better understanding the relationship between COVID-19, pregnancy and poor health outcomes. The first condition, “misattributed dyspnea (shortness of breath)” refers to respiratory symptoms common to both advanced pregnancy and COVID-19, which are mistakenly perceived as related to the former rather than to the latter; as a result, pregnant women with this condition receive no medical attention until the disease is in an advanced stage. The second condition, “silent hypoxia”, refers to abnormally low blood oxygen saturation levels in COVID-19 patients, which occur in the absence of typical respiratory distress symptoms, such as dyspnea, thereby also leading to delayed diagnosis and treatment. The delay in diagnosis and referral to treatment, due to either “misattributed dypsnea” or “silent hypoxia”, may lead to rapid deterioration and poor health outcome to both the mothers and their fetuses. This is particularly valid among women during advanced stages of pregnancy as the altered respiratory features make the consequences of the disease more challenging to cope with. Studies have demonstrated the importance of monitoring blood oxygen saturation by pulse oximetry as a reliable predictor of disease severity and outcome among COVID-19 patients. We propose the use of home pulse oximetry during pregnancy as a diagnostic measure that, together with proper medical guidance, may allow early diagnosis of hypoxia and better health outcomes.

## Introduction

**Figure 1:**
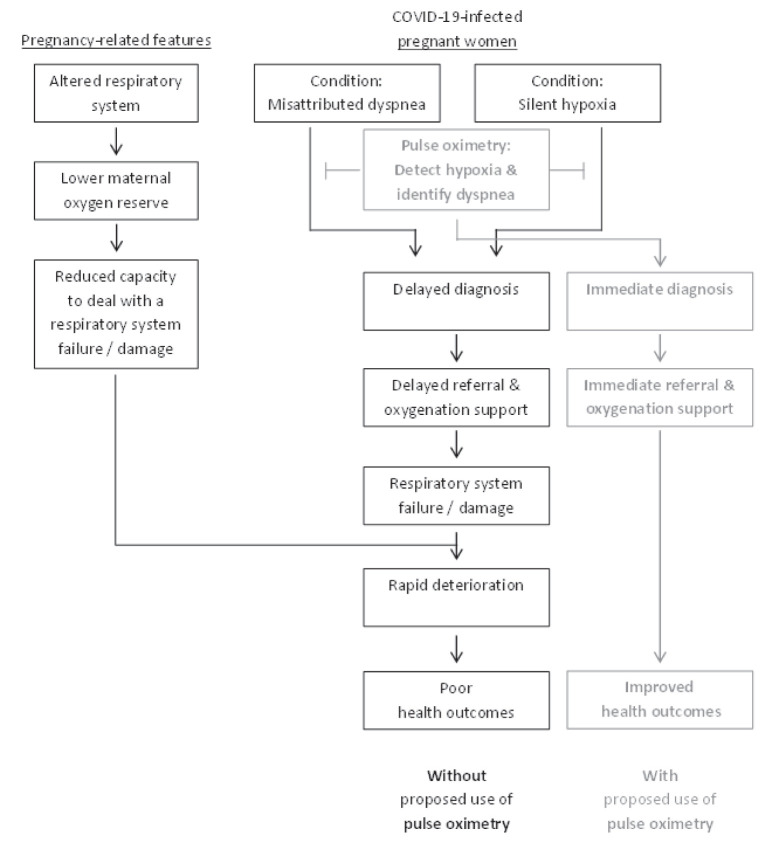
Schematic representation of the conditions contributing to health outcomes in COVID-19-infected pregnant women.

This perspective article deals with the current advances in our understanding of the underlying conditions contributing to poor outcomes of Corona-virus disease 2019 (COVID-19) among pregnant women, and the consequent future directions for policy. To this end, we first provide introduction to COVID-19 and the possible associations with pregnancy. We then focus on the third trimester of pregnancy, specifically relating to natural physiology and metabolic changes and their impact on respiratory function. Then, we put the spotlight on a condition we suggest to term “misattributed dyspnea (shortness of breath)”, namely a common COVID-19 symptom due to hypoxia that coincides with pregnancy-related features, which may be perceived as related to the pregnancy rather than to COVID-19 and hence receive no attention. Later, we describe the phenomena of “silent hypoxia,” which refers to COVID-19 hypoxic patients who do not experience the common respiratory distress symptom of dyspnea. We then describe the implications on medical policy and suggest that pulse oximetry can play a pivotal role in preventing poor health outcomes. These relationships are schematically illustrated in Figure 1.

### COVID-19

A pathogen of the coronavirus clade, that was termed severe acute respiratory syndrome coronavirus-2 (SARS-CoV-2), provoked a global pandemia, which first erupted in mid-late December 2019 in Wuhan city of Hubei province, China, and has been phrased as coronavirus disease 2019 (COVID-19) (1–4). The pneumonia-like outburst and spread throughout the world has posed a serious threat to global health (5), an extensive impact on global trade (6, 7) and the economy (8), and real danger to civic well-being. This contagious viral infection is spread through inhalation of viral droplets, spread as a result of breathing and coughing or even touching infected surfaces (9). Binding between SARS-CoV-2 surface unit of the spike (S) glycoprotein and the cellular carboxypeptidase ACE2 receptor is required for host cell entry (1, 10, 11). The cellular serine protease TMPRSS2 is employed for the Spike protein priming, a cleavage that allows the fusion of viral and cellular membranes (12) and viral spread in the infected host (13). The key player ACE2 is highly expressed in the intestinal tract, kidney, gallbladder, heart, testis and also placental trophoblasts (14)—all shown to be associated with COVID-19 related symptoms—while its expression in the respiratory system is limited. Of note, ACE2 expression is higher in pregnant women, thus they may be at an elevated risk of complications from SARS-CoV-2 infection (15).

The most common clinical signs and symptoms of the disease are fever, fatigue, dry cough and breathlessness, while expectoration, headache, myalgia, diarrhea, nausea, vomiting, loss of taste or smell, cutaneous eruptions and renal failure have also been reported (16–18). Findings showed that majority of patients (~80%) present no or only mild symptoms. Yet, when the virus severely infects and affects person's pulmonary system, it often results in refractory hypoxaemia due to interstitial pneumonia and acute respiratory distress syndrome, leading to extra-pulmonary system dysfunctions and organ damage. In cases of derangements in hematologic and digestive system, sepsis, septic shock and death may occur [for more details on the mechanisms of action underlying disease progression and sevirity see (19, 20) as well as illustrations within].

Risk factors for COVID-19 infection and poor health outcomes—in terms of disease prevalence, severity and/or mortality—include co-existence of cardiovascular and circulatory diseases, diverse respiratory diseases, malignant neoplasms, diabetes, renal failure, sepsis, advanced age and elevated body-mass index (21–23). In addition, marked differences have been observed between women and men (24–27), in favor of women. Nevertheless, pregnant women are at increased risk of infection and severe illness, compared to non-pregnant women.

### Pregnancy and COVID-19

Pregnant women, particularly during the advanced stage of pregnancy, are more susceptible and vulnerable to infection by respiratory pathogens, including COVID-19, and are at a higher risk of severe outcomes (28–31). The accumulating evidence bring to light some COVID-19 major effects on maternal and perinatal outcomes (28, 30, 32). It was claimed that increased rates of certain pregnancy complications among SARS-Cov-2 infected women are observed only in symptomatic COVID-19 (33). Those include higher rates of complications to the fetus and/or mother, mainly in the third trimester [88% of pregnancy complications occur in the third trimester, compared to 9 and 3% in the second and first trimesters, respectively (28)], such as premature rupture of membranes, placenta accreta, premature labor, preeclampsia, cesarean section, fetal distress and perinatal death (32–38). In addition, these pregnant women are at increased risk of severe maternal morbidity (39), specifically the need for hospitalization, intensive care units admission (28, 40), and the need of mechanical respiratory support/invasive ventilation/extracorporeal membrane oxygenation (37, 39–41), and even maternal death (32, 42). Newborns diagnosed with COVID-19 postpartum are reported in some studies. Albeit vertical transmission is mostly uncommon (43), there is evidence that not only placental infection but also fetal infection can occur (44–46). Placentas from infected patients show inflammatory, thrombotic, and vascular changes which in turn could also potentially result in long-term, multi-systemic implications in exposed infants (47). A third trimester stillbirth study suggested increased placental thrombotic pathology during the COVID-19 pandemic (48).

Analysis of mechanical, physiological and immunological changes during pregnancy, and particularly in the second and third trimesters, can explain the causes of higher susceptibility, vulnerability and poor prognosis—including hypoxic compromise—of infected pregnant women and their newborns (30, 49).

In many aspects, severe COVID-19 may be regarded as a viral-induced hyper-inflammatory condition with multi-organ involvement due to a cytokine cascade (50). Pro-inflammatory cytokine interleukin-6 (IL-6) and C-reactive protein (CRP) are biomarkers associated with the COVID-19 progression, severity and mortality (51). Elevated IL-6 (52, 53) and CRP (36, 37, 54) circulating levels are closely related to COVID-19 disease severity, and were shown to be higher also among pregnant women (55) and to predict a need for mechanical ventilation among COVID-19 patients (56). It is possible that the SARS-CoV-2-induced cytokine storm may result in a more severe inflammatory state mainly in third trimester pregnant women, as they are already in a pro-inflammatory state, which is required for preparation toward childbirth (57).

These lines of evidence may suggest that respiratory failure among COVID-19 patients is associated with changes in cytokine profiles, such as over-production of IL-6 and CRP, which can be intensified during the second and mainly the third trimesters of gestation. What physiological processes occur in the third trimester, mainly with regard to the respiratory process and function, and how do these processes relate to COVID-19?

### Pregnancy and Physiology of Cardiorespiratory System in the Third Trimester

According to the World Health Organization (WHO) data, the growth in estimated fetal weight during pregnancy is not linear, where fetal weight grows in accelerating rate as pregnancy progresses, becoming more pronounced after mid-second trimester (58, 59).

Physiological and metabolic changes during pregnancy (60), and particularly during advanced stages, have a significant impact on the immune system, respiratory system, cardiovascular function, and coagulation, which may affect the mother as well as COVID-19 disease progression (28). Modifications to pulmonary function, ventilatory pattern and gas exchange are of particular relevance to this paper.

Pregnancy may be viewed as a condition of immune suppression, affecting the outcome of the pregnant woman's susceptibility to and severity of certain infectious diseases. While pregnant women respond like non-pregnant women to some infectious states, they are more susceptible to other infections (61, 62). Pregnancy is characterized by immune tolerance to the fetal-placental unit as a transplant (63), and by attenuation of some autoimmune diseases (64).

Physiological and biochemical alterations of hormonal patterns (progesterone, estrogen, prostaglandins) are the main cause of ventilatory changes in respiratory function, as well as mechanical pathways which affect lung volume, spirometry, airway function, chest wall geometry and displacement, respiratory muscles and breathing patterns (65, 66).

For instance, the progressive uterine distension during pregnancy is the major cause of lung volume reduction and chest height shortening, which in turn lead to an elevation of the diaphragm (its resting position moves upward) and alter the thoracic configuration. This leads to an earlier closure of the small airways accompanied not only by abdominal enlargement but also by ribcage dimension increase [for detailed description see (65, 66)]. These processes become more pronounced and impactful as pregnancy progresses, mainly during the second half of pregnancy. As a result, expiratory reserve volume (ERV), functional residual capacity (FRC) and residual volume gradually decrease, by up to 40% at term, by 17–25% (67), and by 7–22% [reviewed in (65)], respectively. Inspiratory capacity increases at the same rate in order to maintain stable total lung capacity (TLC). Furthermore, minute ventilation increases [by almost 50% (68)] during the first trimester of gestation (and maintained throughout the course of pregnancy), due to higher tidal volume with unchanged respiratory rate (65). Also, oxygen consumption increases [by 21–35% (69)] as pregnancy progresses (65, 70). Consequently, these modifications lower the oxygen reserve of pregnant women.

There is conflicting evidence concerning arterial oxygen saturation during pregnancy. Some reports claimed that not only are blood oxygen saturation (SpO_2_) values of pregnant women lower than those of non-pregnant woman (71), saturation level decreases (though not appreciably) as pregnancy progresses, while respiratory rate remain unchanged and heart rate rises (72). However, other reports argued that the arterial oxygen saturation does not show any statistically significant difference in pregnant women as compared to non-pregnant women (73), and that findings about its level decrease throughout pregnancy are not conclusive (70).

All the physiological alterations during pregnancy are required to satisfy the increased maternal oxygen demands, due to heightened metabolism, gestational anemia (if induced), and fetal oxygen consumption. These increased oxygen demands are usually accompanied by dyspnea, which is also a common symptom of both hypoxemia and COVID-19.

### Hypoxia, Silent Hypoxia, and COVID-19

Hypoxia refers to the state of a reduced level of oxygenation in the body's tissues. This state can be due either to ineffective delivery of oxygen to the tissues and/or to ineffective utilization of oxygen by the tissues. Hypoxemia is defined as a decrease in the blood oxygen level, namely reduced arterial oxygen tension/partial pressure (74, 75). Hypoxemia can lead to hypoxia. Although hypoxemia and hypoxia refer to different conditions which do not always coexist, these terms are used interchangeably. Normally, hypoxia leads to dyspnea, rapid breathing, fast heart rate, and other symptoms (75, 76).

Silent hypoxia (also called asymptomatic, happy, and apathetic hypoxia as well as silent hypoxemia) refers to the condition in which patients have relatively well-preserved lung compliance and a severely compromised pulmonary gas exchange, leading to grave hypoxia/hypoxemia, yet without proportional signs of the common respiratory distress symptoms of dyspnea (77–81).

This novel condition has been described as “a mismatch [between] what we [physicians] see on the monitor and what the [COVID-19] patient looks like in front of us” (82), and as “a dissociation between the relatively well-preserved lung mechanics and the severity of hypoxemia” (83). One can view silent hypoxia, hypoxia-induced gasping, and the death zone as pathophysiological stages of the progressive condition in COVID-19 (84). The novel clinical phenomenon of silent hypoxia in COVID-19 patients contrasts with the experience of physicians who usually treat critically-ill patients in respiratory failure. Thus, proper recognition of the threat is challenging (85, 86), and consequently referral of patients to the intensive care unit for supplementary oxygen is often delayed (87, 88).

Silent/asymptomatic hypoxia occurs in more than 50% of all severely-ill COVID-19 patients (77, 89), mainly observed among patients admitted for severe COVID-19 (90). Silent hypoxia is actually considered as the underlying cause of rapid clinical deterioration and mortality in the hospital setting, due to delayed patient arrival, delayed diagnosis and delayed administration of appropriate care (91, 92). It is associated with poor health outcomes in hospital settings (87, 89, 92) as well as at home (89). So far, the frequency of silent hypoxia among COVID-19 infected pregnant women has not been reported.

The pathophysiology of silent hypoxia, relating to respiratory and diverse neurological components (78, 85, 93–97), as well as modeling of the lung perfusion abnormalities to explain this phenomenon (98), have been recently described, yet this topic is out of the scope of this paper.

Of special note, COVID-19-induced hypoxia may affect the placental oxygen supply and cause severe complications to fetuses and newborns of infected pregnant women (49), as hypoxia can play a significant detrimental physiological role in fetal development, in regard to placentation, angiogenesis, hematopoiesis, fetal programming and the function of diverse organs (99).

### Misattributed Dyspnea and Silent Hypoxia During Pregnancy

Considering the physiological and metabolic changes during advanced stages of the pregnancy, and their impact on the immune system, respiratory system and cardiovascular function, COVID-19 infected pregnant women are more physically compromised and less able to cope with the disease in general and particularly with the consequences of delayed diagnosis and overdue referral to treatment, compared to non-pregnant women. We propose that these may occur due to two main conditions: **misattributed dyspnea** and **silent hypoxia**.

Our perspective posits that among COVID-19 infected pregnant women, symptoms such as dyspnea may be attributed to pregnancy, rather than to COVID-19, and therefore are often not further pursued. Therefore, we propose the term “misattributed dyspnea” to describe the condition where the COVID-19 symptom of dypsnea is mistakenly perceived as one of the symptoms of “normal” advanced pregnancy. This condition can lead to the disease not being properly and timely diagnosed, monitored and treated, leading to rapid deterioration. Of note, a similar phenomenon was proposed regarding late pregnancy estrogen-induced gestational rhinitis and the coryzal symptoms of COVID-19 (30).

Our perspective also posits that the well-established phenomenon of silent/asymptopmatic hypoxia can also occur among pregnant women with COVID-19. This condition has been coined “silent” due to its ability to quietly inflict damage to COVID-19 patients; in other words, it can cause parts of the lungs to be incapable of functioning properly, without the patient being able to detect this effect. Abnormally low oxygen levels in the body can irreparably damage vital organs, if gone undetected for too long.

Interestingly, a recent thematic analysis of over 700 PFD (Prevention of Future Death) coroners' reports in England and Wales, during a 3-year period (2016–2019), revealed that “missed, delayed or uncoordinated care” is a major cause of death in health care settings (100). This primary theme is central to both health care in general (100) and in particular to our proposed hypoxia-related conditions underlying poor COVID-19 outcomes among pregnant women.

The two conditions, misattributed dyspnea and silent hypoxia, present additive serious challenges to pregnant women with COVID-19, and may provide a novel explanation for the underlying causes of the disease's rapid progression and poor outcomes. Moreover, clinical implications may pave the way toward correct and timely diagnosis and personalized treatment, for pregnant women with COVID-19.

## Implications

The following policy implications focusing on early detection of hypoxia, mainly the silent form, due to its central place in COVID-19 disease prognosis and outcomes, as well as differentiating dyspnea symptom related to pregnancy from that related to hypoxia induced by COVID-19.

**(1) Increasing awareness** – Increasing the awareness of both pregnant women and the medical care staff to the relevant phenomena—dyspnea, misattributed dyspnea, hypoxia, and silent hypoxia—and specifically relating these phenomena to the shared features of pregnancy and COVID-19, may significantly improve diagnosis, monitoring, treatment and healthy outcomes. The clinical guidelines regarding the follow-up of COVID-19 infected pregnant women and their newborns has been proposed (30).
**(2) Clinical management** – Medical staff need to be introduced to a clinical management protocol where “aspects specific to COVID-19 and gestation should be known by specialists in order to correctly diagnose the disease, classify the severity, distinguish specific signs of COVID-19 from those of obstetric complications, and take the most appropriate management decisions” (101). The medical staff also need to be introduced to a management protocol for subclinical hypoxemia in COVID-19 patients (91, 102), so that they can proactively apply this protocol to pregnant women, either suspected or confirmed of being infected with COVID-19, even if these women do not complain or present “additive” symptoms to their regular pregnancy-related course. The protocol would require pregnant women infected with COVID-19, which are mostly not aware of the disease progression, to be checked for hypoxia at medical centers and at home.
**(3) Self-monitoring** – We suggest that pulse oximeters be distributed to pregnant women, in order for them to self-monitor and identify low blood oxygen levels. Guidance regarding the oximeters' proper use and when patients should seek medical care is required. During the COVID-19 pandemic, such actions are relevant, justified and required not only of pregnant women but also of other populations at risk. A practical guide for remote management of COVID-19 using home pulse oximetry was recently published (103). In the following sections, we elaborate on the importance of pulse oximentry to COVID-19 management, and deliberate on policy-related aspects.

### Pulse Oximeters and COVID-19

It has been proposed that the pulse oximeter should be as omnipresent as the thermometer in public health care systems, especially in the COVID-19 era (104). Pulse oximetry is a key biomarker for early identification of hypoxia among COVID-19 patients (105–107) and as a means to predict COVID-19 outcomes in patients with silent hypoxia (89). Furthermore, identifying hypoxia among non-severe COVID-19 patients, including those discharged from emergency rooms or in outpatient testing centers, can also insure better health outcomes. It was argued that silent hypoxia “requires early and aggressive implementation of home- or community-based pulse oximetry programs, combined with around-the-clock telemedicine services, to effectively intercept patients who may be entering the rapid deterioration phase of COVID-19” (102, 108).

Home pulse oximeter monitoring can (a) identify the need for hospitalization in initially non-severe COVID-19 patients; (b) reduced unnecessary emergency department revisits (109); and (c) considerably improve patient outcomes reducing the odds of longer length hospital stays and mortality (110). For example, oximetry monitoring has played a central role as part of intensive contact tracing, leading to a reduction in COVID-19 mortality rate by ~50% among a high-risk population, the White Mountain Apache tribe in rural Arizona (111). Moreover, the lowest pre-hospital recorded values for oxygen saturation levels independently predict death in COVID-19 patients (112). A recent retrospective study based on data from a large South African insurance company showed that high-risk COVID-19 patients who routinely used a home pulse oximeter had a statistically significant lower mortality rate than patients who did not do so (113).

### Pulse Oximeter Reading and Policy

Importantly, a disparity in oxygen provision for COVID-19 patients between different countries was recently demonstrated, and a strong inverse correlation has been demonstrated between the national thresholds for the commencement of oxygen support and national case fatality rates (114). Therefore, an examination and re-assessment of national thresholds for the commencement of oxygen support, adjusted to the COVID-19 pandemic, are justified.

### Practicalities and Cautions Related to Pulse Oximetry Use

It is important to take into account possible inaccurate readings of some of the low-cost pulse oximeters (115), the intrinsic and extrinsic influences on pulse oximetry values (116, 117), and the effect of dark skin color, sensor type and gender (118, 119) on oximeter accuracy. Open questions, alongside key success factor, were described in details elsewhere (103).

### Recommendations and Guidelines

Recently, the World Health Organization published a conditional recommendation for the use of pulse oximetry monitoring at home as part of a package of care, though it is aimed at COVID-19 symptomatic patients with risk factors for progression to severe disease who are not hospitalized (https://app.magicapp.org/#/guideline/j1WBYn/rec/nyo9Zj). A useful practical guidance for the correct use of home pulse oximeter for monitoring patients with COVID-19 is now available (120).

In spite of the accumulating evidence on the effectiveness of home pulse oximetry monitoring by COVID-19 patients, this practice has not been widespread. Remote home monitoring models using pulse oximeters have been implemented for confirmed or suspected COVID-19 cases in only few countries [reviewed in (121)]. We would like to see more widespread implementation at the international and national levels. Accumulating evidence suggest that such implementation, combined with public education and guidance as described before, may serve as useful preventive means for pregnant woman with COVID-19.

## Concluding Remarks

In summary, our perspective is that the recognition and treatment of hypoxia in the pregnant COVID-19 patient can be delayed—with dangerous consequences—in two ways: (1) misattribution of symptoms of dyspnea as a typical manifestation of pregnancy, rather than as a progression of pulmonary disease related to COVID-19, and (2) silent hypoxia, where symptoms typical of hypoxia (such as dyspnea) do not occur until it is “too late.” These two scenarios can and should be detected early with widespread use of home oximeters for all pregnant women confirmed or suspected COVID-19, paving the way toward breaking the link between advanced pregnancy and poor COVID-19 health outcomes. This practice should also be applied first to other populations at risk and then to all COVID-19 patients.

## Data Availability Statement

The original contributions presented in the study are included in the article/supplementary material, further inquiries can be directed to the corresponding author/s.

## Author Contributions

YM envisioned and conceived the fundamental basis of this manuscript, which was further developed collaboratively by all authors. All authors listed have made a substantial, direct and intellectual contribution to the work, and approved it for publication.

## Conflict of Interest

The authors declare that the research was conducted in the absence of any commercial or financial relationships that could be construed as a potential conflict of interest.

## Publisher's Note

All claims expressed in this article are solely those of the authors and do not necessarily represent those of their affiliated organizations, or those of the publisher, the editors and the reviewers. Any product that may be evaluated in this article, or claim that may be made by its manufacturer, is not guaranteed or endorsed by the publisher.

## Abbreviations

COVID-19: coronavirus disease 2019
CRP: C-reactive protein
ERV: expiratory reserve volume
FRC: functional residual capacity
IL-6: Interleukin-6
SARS-CoV-2: severe acute respiratory syndrome coronavirus-2
TLC: total lung capacity
WHO: World Health Organization.
